# Above and below-ground growth, accumulated dry matter and nitrogen remobilization of wheat (*Triticum aestivum*) genotypes grown in PVC tubes under well- and deficit-watered conditions

**DOI:** 10.3389/fpls.2023.1087343

**Published:** 2023-03-07

**Authors:** R. Rustum Zhiipao, Vijay Pooniya, Dinesh Kumar, Niraj Biswakarma, Yashbir Singh Shivay, Anchal Dass, Naresh Kumar Bainsla, Kamlesh K. Lakhena, Rakesh K. Pandey, Anamika Burman, Arti Bhatia, Ram D. Jat, Prabhu Govindasamy, Karivaradharajan Swarnalakshmi, Kajal Das, Ram L. Choudhary, Subhash Babu

**Affiliations:** ^1^ Agronomy, ICAR-Indian Agricultural Research Institute (IARI), New Delhi, India; ^2^ Agronomy, G.D. Goenka University, Gurugram, Haryana, India; ^3^ Genetics, ICAR-Indian Agricultural Research Institute (IARI), New Delhi, India; ^4^ Plant Physiology, ICAR-Indian Agricultural Research Institute (IARI), New Delhi, India; ^5^ Environmental Science, ICAR-Indian Agricultural Research Institute (IARI), New Delhi, India; ^6^ Agronomy, Chaudhary Charan Singh Haryana Agricultural University (CCSHAU), Hisar, Haryana, India; ^7^ Microbiology, ICAR-Indian Agricultural Research Institute (IARI), New Delhi, India; ^8^ ICAR-Central Research Institute for Jute and Allied Fibers, Barrackpore, West Bengal, India; ^9^ ICAR-Directorate of Rapeseed-Mustard Research, Bharatpur, Rajasthan, India

**Keywords:** wheat genotypes, dry matter accumulation, root -traits, nitrogen partitioning, water use efficiency

## Abstract

The continuing decline in water resources under the ever-changing climate compels us to re-orient our focus to a more sustainable practice. This study investigates the performance of *Triticum aestivum* wheat genotypes viz. HD-2967, HD-3086, HD-3249, DBW-187, and HD-3226 under well- and deficit-watered conditions for their root-traits, biomass and nitrogen accumulation and remobilization, and water use efficiencies, grown in PVC-tubes. The genotypes HD-2967, HD-3086, HD-3249, DBW-187, and HD-3226 under well-watered (WW) resulted in 36, 35, 38, 33, and 42% more grain yield compared to deficit-watered (DW). Among the genotypes, HD-3249 had the highest grain yield under both well- and deficit-watered conditions. Compared to DW, the WW had 28%, 30%, and 28% greater root length, biomass, and root length density at flowering {102 days (d), Z_61_}, while among the genotypes, HD-3249 had relatively greater root-traits. At flowering (Z_61_) and maturity (132 d, Z_89_), genotypes under WW accumulated 30-46% and 30-53%, respectively greater shoot biomass over the DW. Furthermore, the shoot biomass remobilised for HD-2967, HD-3086, HD-3249, DBW-187, and HD-3226 under the WW was 32, 37, 39, 35, and 35% greater than the DW. The nitrogen partitioning to different plant parts at flowering (Z_61_) and maturity (Z_89_) was significantly greater with the WW than with DW. The total nitrogen- remobilized and contribution to grain-N under the WW was 55, 58, 52, 53, 58% and 9, 19, 15, 17, 17% greater than the DW for the genotypes HD-2967, HD-3086, HD-3249, DBW-187, and HD-3226. The irrigation water use efficiency (WUE) at flowering (Z_61_) was more under the deficit-watered, but the biomass and grain total WUE was improved with the well-watered condition. Hence, it is apparent that proper scheduling of irrigation and N applications, along with the adoption of a genotype suited to a particular environment, will result in better WUE and grain yields, along with better utilization of scarce resources.

## Introduction

Wheat (*Triticum aestivum* L.), is the most widely grown cereal, being next to rice in India. Since the inception of the green revolution, wheat yields and productivity have been in an upward trend, particularly in the irrigated agro-ecologies. Nevertheless, with the ever-increasing climate change coupled with the rapid depletion of ground water due to the widespread over-extraction (Zaveri et al., 2016; Dangar et al., 2021), the sustainability of wheat production is being threatened. The ground water levels in the major wheat production belt of the north-western India have dropped from 8 mbgl (meters below ground level) to 16 mbgl since 1980, while it was 1 to 8 mbgl in the rest of the country (Rodell et al., 2009; Aeschbach-Hertig and Gleeson, 2012). Furthermore, the declines in the ground water could lead to the poverty elevation (Sekhri, 2014) and threaten the food production, especially by the small-scale farmers who are already poor (Singh et al., 2002). In addition, the fast-declining ground water (major source for irrigation) could challenge the realization of wheat production potential in the near future. Hence, irrigation is crucial for the continued yield enhancement of wheat, particularly the semi-arid region of Indo-Gangetic plains and other similar ecologies, wherein it is grown during the winter (post-monsoon) with negligible and uneven distribution of rainfall. Thus, the need of the hour and years ahead under the changing climatic conditions is to concoct varied management strategies for efficient and productive utilization of the depleting resources, particularly water.

The primary wheat production have been limited due to the shortage of water (Austin, 2011; Jha et al., 2017), wherein the dry matter accumulation and yield are dependent on the optimum and timely availability of water (Kang et al., 2002). One of the most considered environmental stresses is drought (Cattivelli et al., 2008), which is reported to have reduce the wheat yield up to 50% (Reynolds et al., 2007), mainly through the significant reduction in the growth and shoot production (Ehdaie et al., 2012). Subsequently, there is an adverse effect of the drought on the number of effective tillers, leaf area and photosynthesis, and leaf senescence (Gajri and Prihar, 1983). Furthermore, the water stress before anthesis in wheat have been reported to reduce the number and size of spike, while stress during the early grain development curtails the sink potential of grain by shortening the grain filling duration (Saini and Westgate, 1999). The wheat crop in the dry land areas for grain filling have higher dependency on the stem reserves rather than the current photosynthesis (Ehdaie et al., 2006), indicating the importance of the enhanced food reserves capability of stem and spike for supporting the grain filling (Blum, 1998; Xue et al., 2006). The contribution of the pre-anthesis assimilate in wheat stem and sheath is normally 25-55% of the final grain weight (Gebbing and Schnyder, 1999), but under water stress condition its contribution increased up to the 80% (Blum et al., 1994Xue et al., 2006). The reduction in the source promotes the reallocation of the reserve photosynthates to the grain, by decreasing the dry matter allocation to the stem and sheath (Wang et al., 1997). Yang et al. (2001) reported that the drought stress at the post-anthesis led to reallocation of 75-92% pre-anthesis carbon stored in the straw to the grain.

Nitrogen (N) play a vital role in wheat, affecting productivity, nitrogen and water use efficiency (Zhou et al., 2011; Grant et al., 2012), wherein with the optimum supply the yield increases (Yang et al., 2017). Studies have shown that under the deficient water condition, the N- absorption and use efficiency decreased and subsequently the grain yield (Plaut et al., 2004). In contrast, the above-ground N uptake, evapotranspiration, and grain yield of wheat increases with the increasing rate of irrigation supply, but the water-use efficiency gets reduced (Zhang et al., 2006). The vital processes for determining the grain quality and yield are N accumulation and remobilization (Hirel et al., 2007; Gaju et al, 2011). In wheat, it has been reported that the remobilization of the N stored in shoots and roots before anthesis contributes 50-95% of the total grain N at maturity (Palta and Fillery, 1995; Kichey et al., 2007). Further, the ability to store higher amount of pre-anthesis N in the stem internodes could enhance the remobilization of larger N to grains, without affecting the photosynthetic capacity (Bertheloot et al., 2008; Foulkes et al., 2009).

The hidden half of the plant (roots) is critical for the water and nutrient uptake, though it contributes only 10-20% of the total plant weight but greatly influence the growth and final yield of the crops (Fageria, 2005; Doussan et al, 2006). A well-developed root system could enhance the absorption capacity of water and nutrients which makes it more opportune for yield increment and use efficiency of the water and N (Palta et al., 2007). The physiology and morphology of root have a close association with the uptake of soil nutrients and water, henceforth affects the shoot growth and development (Ju et al., 2015). Irrigation regimes strongly influenced the depth and density of root by influencing the soil water content (Li et al, 2010), causing the variation of winter wheat grain yield (Kang et al., 2000). Li et al (2010) have reported that the vertical root penetration increases with the slight shortages of water during the vegetative stage causing an increase in deeper layers root length density (RLD). In addition, Rasmussen et al. (2015) found that application of moderate quantities of N in winter wheat promote root growth and enhanced root density coupled with root growth differences among the cultivars. Furthermore, it has been reported that water and N stressed always result in higher root:shoot ratio as the adverse effect on root growth was lower compared to above-ground growth (Zhang et al, 2009). Indeed, Zhang et al (2009) and Ma et al. (2010), reported that there is a tendency for higher grain yield and water use efficiency from crops having low root:shoot ratio, due to greater partitioning of dry matter to the above -ground biomass.

This PVC tube study had the following objectives (i) to assess the genotypic differences of yield, root growth, water use efficiency, and (ii) dry matter and N accumulation, distribution, and remobilization under well- and deficit-watered conditions.

## Materials and methods

### Growth conditions

The present study was carried out during the winter 2021-22 (November-April) in a naturally ventilated net-house and the crop was grown in a PVC-tubes. In all the experimental units, soil and weather conditions were uniform. We closely monitored the experiments throughout the growing season and recorded above and below-ground parameters at different crop cycle intervals. The climate of the region is semi-arid and falls under the trans Indo-Gangetic plains (28°38′ N latitude, 77°10′ E longitude, and 229 m above mean sea level). The mean maximum and minimum temperatures during the crop growth ranged between 17-38°C and 7-17°C, respectively, and received 181.5 mm of rainfall (**Supplementary Figure 1**). The plants were grown in PVC tubes with the two irrigation schedules; (i) well-watered (WW) and (ii) deficit-watered (DW). There were three replicates for each treatment and the tubes were arranged randomly. The plants were grown in a 25 mm PVC tube having 19.5 cm diameter, and two lengths (depths), i.e., 50 cm and 100 cm, wherein tubes of 50 cm length were used for first sampling and 100 cm length for the second sampling. A cylindrical and transparent polyethylene sheet was placed inside each PVC tube, with perforated bottom. A thin layer of pebbles was placed on the bottom for the natural drainage to avoid the accumulation of excess water.

The tubes were laid vertically on the steel stands, filled with soil to the depth of 50 cm and 100 cm, respectively. The soil was sandy-loam in texture and had pH 8.1 (1:2.5, soil: water), organic carbon 4.02 g kg^-1^ (Walkley and Black, 1934), KMnO_4_-oxidizable N 117.6 mg kg^-1^ (Subbiah and Asija, 1956), 0.5N NaHCO_3_-extractable P 6.69 mg kg^-1^ (Olsen, 1954), and 1N NH_4_OAc- exchangeable K 123 mg kg^-1^ (Hanway and Heidel, 1952). The soil for filling these tubes was collected from the adjoining field (0-0.15 m depth), air dried, sieved to 5 mm and packed carefully into the tubes, to a bulk density of approximately 1.46 g cm^-3^. The soil filled tubes were manually watered carefully to saturation and avoid the excessive drainage. Five pre-germinated seeds each of the five wheat genotypes were dibbled manually once the soil in the tube attained the field capacity on 24 November 2021. Subsequently, thinning operation was done at 3-4 leaf stage leaving one healthy plant in each tube. Water application schedule for (i) well-watered: crown root initiation (25 d, 1.5 L), active tillering (45 d, 1.5 L), flowering (102 d, 1.5 L), and grain filling (115 d, 1.0 L); (ii) deficit-watered: crown root initiation (1.5 L), flowering (1.5 L), and grain filling (0.5 L). The plants were manually watered as per the above schedule.

Nutrients were added with an equivalent rate of 120 kg N ha^-1^ (urea, CH_4_N_2_O), 26.2 kg P ha^-1^ (diammonium phosphate, (NH_4_)_2_HPO_4_), and 33.3 kg K ha^-1^ (muriate of potash, KCl), which is considered as the recommended fertilizer rate for wheat in the region. For the deficit-watered tubes, ½ N along with full dose of P and K was mixed in the top 10 cm at sowing, while the remaining ½ N was applied at the crown root initiation stage. In well-watered tubes, ½ N along with the full dose of P and K was applied at sowing and the other ½ N in two equal splits was applied at the CRI and active tillering stages of the crop.

### Planting material

Five *Triticum aestivum* L. wheat genotypes viz., HD-2967, HD-3086, HD-3249, DBW-187, HD-3226 of recent years widely grown in Indian sub-continent under timely sown irrigated conditions were used for the study. In brief, the HD-2967, HD-3086, and HD-3226 genotypes were released for the north-western Indo-Gangetic plains, with an average yield of 5.4 Mg ha^-1^, 5.4 Mg ha^-1^, and 5.7 Mg ha^-1^, respectively and maturing in 140 d. While, HD-3249 and DBW-187 for north-eastern Indo-Gangetic plains with a mean yield of 4.8 Mg ha^-1^ and 5.7 Mg ha^-1^, respectively and maturing in 125 d. The HD-series were developed by the ICAR-Indian Agricultural Research Institute, and the DBW-187 by the ICAR-Indian Institute of Wheat and Barley Research. These genotypes had a good disease resistance against the rust/Karnal bunt apart from having excellent quality parameters for varied usage.

### Plant sampling, measurements and analysis

**Figure 1:**
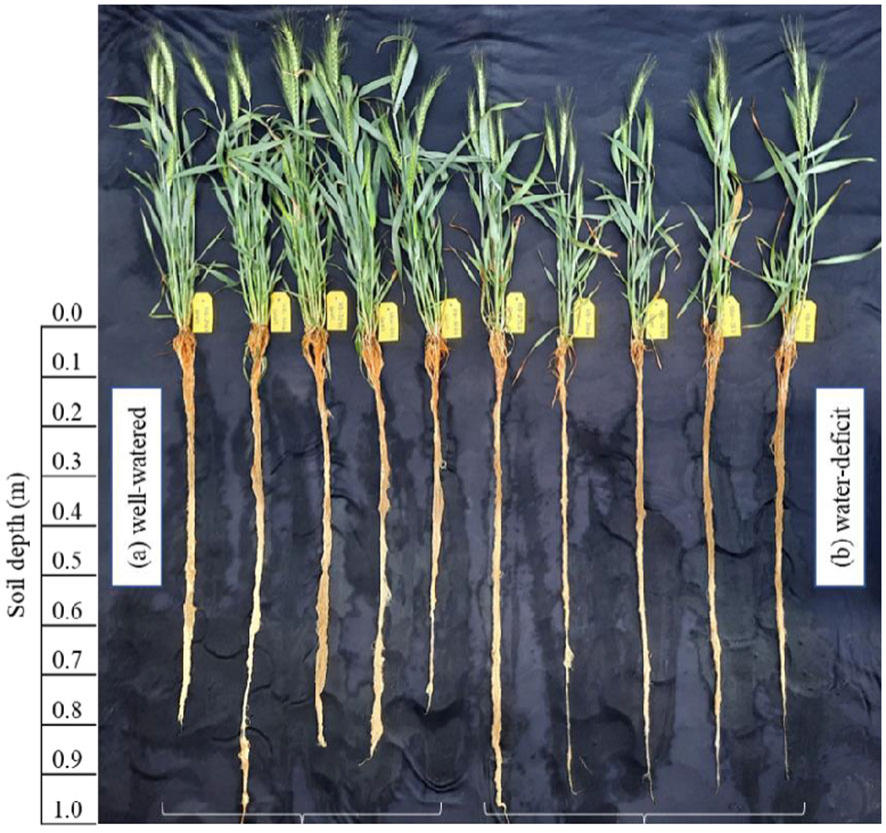
Destructively measured root growth pattern of the five timely sown wheat genotypes (HD 2967, HD 3086, HD 3249, DBW 187, HD 3226) grown under well-**(A)** and deficit **(B)** watered conditions. Genotypes were sampled at the flowering (Z61), 102 days of sowing.

The plant phenology was monitored using Zadoks et al. (1974) scale. Plants were assessed at 45 d (tillering stage, Z_25_), 102 d (flowering, Z_61_), and 132 d (maturity, Z_89_) with three tubes from each treatment. At first (45 d) and second (102 d) sampling, the cylindrical transparent polyethylene sheet placed on the inner PVC tube was gently pulled, placed in still water till saturation and repeatedly washed carefully to remove the attached soil (**Figure 1**). The above-ground plant parts and whole roots were excised at the root/shoot interface, separated into leaf, stem + sheath (first sampling), and flag leaf, stem + sheath, leaf (excluding flag leaf), and spike (second sampling). The leaf area per plant was recorded using the leaf area meter (Model LICOR–3100).

The plant parts were oven dried at 65 ± 5 °C until obtaining the constant weight, and the dry weight of each part was recorded separately. Subsequently, the washed roots were placed in the plastic bags and kept at 5 °C. Roots were then scanned and the images of the roots were analyzed with WinRHIZO professional software (LA2400, Regent instrument, Quebec, Canada). Thereafter, the scanned roots were dried in a hot air oven at 65 ± 5 °C to record the root biomass. The root length density was computed by dividing the total root length with the soil volume (0.0149 m^3^- first sampling; 0.0298 m^3^- second sampling). While, the root: shoot ratio (R:S) was calculated by dividing the root biomass by shoot biomass (Pooniya et al., 2020). At maturity, exposed peduncle length, effective tillers (tillers bearing the spike), spike length, and spikelets spike^-1^ for each genotype and treatments were recorded. The harvested plants were separated into stem (including sheath), leaves, flag leaf, and spike (glumes + awns + grain), and oven-dried at 65 ± 5 °C to the constant weight. The oven dried spikes were manually threshed, separated into the grain and glumes + awns, weighed, milled and stored for the nutrient analysis. Dry matter and nitrogen content in different plant parts were measured at flowering and maturity stages, while phosphorus and potassium contents were measured only at maturity stage. The appropriate amount (0.5 g) of the ground samples for different plant parts were used to determine the total N concentration employing the modified Kjeldahl digestion method, total P by colored Vanado-molybdo-phosphoric acid method, and total K by the flame photometer method (Prasad et al., 2006). The nutrient uptake was obtained by multiplying the respective concentration in different plant parts with their corresponding dry weight. The leaf relative water content (RWC) at flowering was calculated as suggested by Turner (1981).

RWC (%) = [(FW-DW)/(TW-DW)]*100

Wherein, FW is fresh weight, DW is dry weight, and TW is turgid weight

Dry matter remobilization amount and contribution of remobilised DM to grain yield vis-à-vis nitrogen remobilization and remobilised N contribution to grain N was calculated as follows (Papakosta and Gagianas, 1991; Ntanos and Koutroubas, 2002; Zhang et al., 2017).

1) Dry matter remobilization (DMR, g plant^-1^) = Dry matter in the crop component at anthesis – Dry matter in the crop component at maturity
2) DMR contribution to grain yield (%) = (DMR/grain yield) × 100
3) Nitrogen remobilization (N_R_, mg plant^-1^) = Nitrogen content in the crop component at anthesis – Nitrogen content in the crop component at maturity
4) N_R_ contribution to grain N (%) = (N_R/_grain N) × 100

The biomass (at maturity, only vegetative parts were considered) and grain water use efficiency (Qiu et al., 2008; Liu et al., 2018) was calculated using the following formulas:

*1) Irrigation water use efficiency (WUE_i_, g L^-1^ plant^-1^) = Dry matter or grain yield/amount of water applied*

*2) Precipitation water use efficiency (WUE_p_, g L^-1^ plant^-1^) = Dry matter or grain yield/amount of rainfall received*

*3) Total water use efficiency (WUE_t_, g L^-1^ plant^-1^) = Dry matter or grain yield/amount of water applied + rainfall received*

### Statistical analysis

The data was subjected to two-way ANOVAs with SAS 9.4 to test the main effects (water and genotypes) and their interactions. Tukey’s HSD at 0.05 probability separated the mean effects of the treatments. The Pearson correlation analysis examined the association between different variable.

## Results

### Yield and yield traits

Water application had the significant differential impacts on the yield and yield traits of wheat genotypes (**Table 1**) grown in a PVC-tubes. The well-watered (WW) treatment had yield advantage of 36%, 35%, 38%, 33%, and 42% for HD-2967, HD-3086, HD-3249, DBW-187, and HD-3226, respectively over the deficit-watered (DW). Among the genotypes, DBW-187 had the least yield reduction while the highest reduction in yield was recorded with HD-3226. Under WW, HD-3226 recorded the greatest effective tillers, which was significantly higher than HD-3086. The exposed peduncle length was the highest with the HD-3086, wherein it was similar to HD-3249 under the WW, but significantly longer than other genotypes in both the water treatments. The spike lengths in both the water treatments were comparable among the genotypes except HD-3086 under DW. HD-2967 had the highest number of spikelets spike^-1^ and grains spike^-1^ under WW, and the least was with HD-3086 in DW treatment. Water application didn’t influence the 1000-grain weight (**Table 1**).

**Table 1:** Yielding traits and grain yield of wheat genotypes grown under well- and deficit-watered conditions.

Treatment		Effective tillers	Peduncle length (cm)	Spike length (cm)	Spikelets spike^-1^	Grains spike^-1^	1000-TGW (g)	Grain yield (g plant^-1^)
WW	HD-2967	8.7^a^	8.9^bc^	12.2^a^	20.6^a^	70.8^a^	25.3^f^	12.7^cd^
	HD-3086	5.0^c^	10.9^a^	10.4^ab^	17.8^bcde^	57.2^bc^	34.2^a^	11.4^cd^
	HD-3249	8.3^a^	9.3^ab^	12.4^a^	18.8^bcd^	59.4^b^	33.9^a^	17.1a
	DBW-187	8.0^ab^	7.8^bcd^	11.2^ab^	19.3^ab^	57.0^bc^	28.6^de^	13.3^bc^
	HD-3226	10.0^a^	6.1^de^	12.2^a^	19.5^ab^	54.4^c^	29.0^d^	15.2^ab^
DW	HD-2967	6.0^bc^	8.4^bc^	10.5^ab^	17.2^de^	47.5^d^	28.1^e^	8.1^f^
	HD-3086	4.0^c^	5.4^ef^	9.6^b^	17.0^e^	40.8^e^	32.7^b^	7.4^f^
	HD-3249	6.0^bc^	7.8^bcd^	11.2^ab^	19.0^abc^	58.5^b^	30.7^c^	10.6^de^
	DBW-187	6.0^bc^	7.3^cd^	11.2^ab^	17.3^cde^	46.8^d^	31.1^c^	8.9^ef^
	HD-3226	6.0^bc^	4.1^f^	11.2^ab^	18.0^bcde^	48.2^d^	28.4^de^	8.7^ef^
Water		***	***	*	***	***	ns	***
Genotype		***	***	*	**	***	***	***
Water × genotype		*	***	ns	**	***	***	*

Data are the mean of three replicates for each genotype and water treatments. Values in a column followed by the different letters are significantly different at p<0.05 as determined by Tukey’s honestly significant difference test among the varieties. TGW is thousand grain weight. For ANOVA results, *p<0.05, **p<0.01, ***p<0.001, and ns, not significant.

### Root system traits

**Figure 2:**
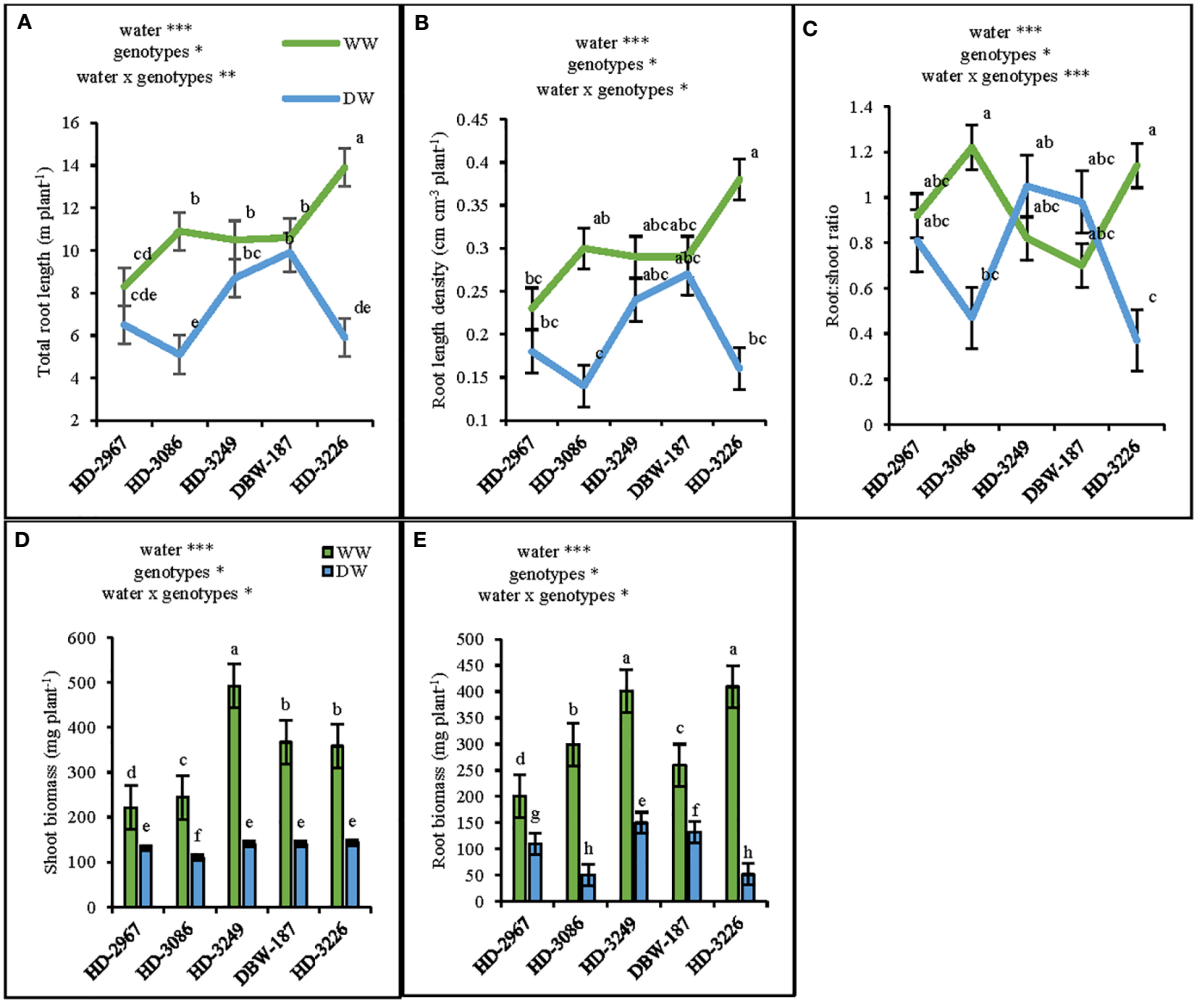
Root-system traits of the five wheat genotypes at 45 days (Z25) under well-(WW) and deficit- (DW) watered conditions. **(A)** total root length, **(B)** root length density, **(C)** root: shoot ratio **(D)** shoot biomass, and **(E)**: root biomass. Letters above the lines and bars indicates significant difference among genotypes at p<0.02(Turkey’s HSD test). Significant at *0.05, **0.05, and ***0.001 probability levels.

**Figure 3:**
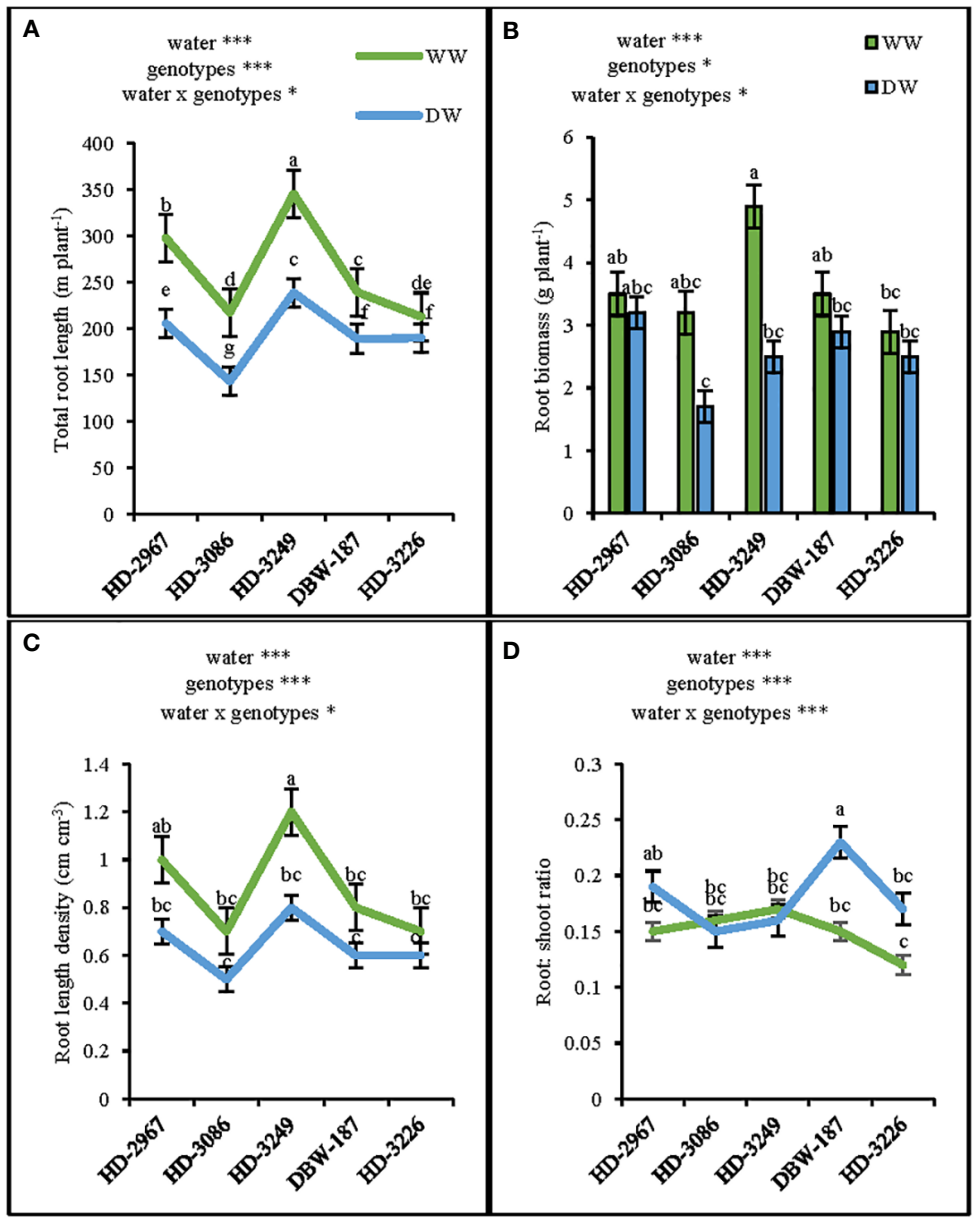
Root-system traits of the five wheat genotypes at flowering (Z61) under well-(WW) and deficit- (DW) watered conditions. **(A)**: total root length, **(B)**: root biomass, **(C)**: root length density and **(D)**: root: shoot ratio. Letters above the lines and bars indicates significant difference among genotypes at p<0.05(Turkey’s HSD test). Significant at *0.05, **0.01, and ***0.001 probability levels.

At 45 d (Z_25_), the shoot biomass under WW and DW was the highest with HD-3249 and HD-3226, respectively. HD-2967, HD-3086, HD-3249, DBW-187, and HD-3226 in WW produced 40%, 54%, 71%, 61%, and 60%, respectively higher shoot biomass over their respective shoot biomass under the DW condition (**Figure 2D**). HD-3226 and DBW-187 had the highest total root length (RL) and density (RLD) both under the WW and DW conditions, respectively (**Figure 2A**). Under WW, the highest root biomass was recorded with HD-3226, while in DW it was HD-3249 and the least among the genotypes was HD-3226 in DW (**Figure 2E**). The root: shoot (R:S) ratio varied largely among the genotypes and water treatment, with the highest R:S ratio recorded with HD-3086 under WW, and least with HD-3226 in DW (**Figure 2C**). The increment in RLD and root biomass (0-50 cm depth) under WW compared to DW for HD-2967, HD-3086, HD-3226, DBW-187, and HD-3226 was 17, 53, 17, 6, 57% and 45, 83, 62, 49, 87%, respectively (**Figures 2B, E**). At flowering (Z_61_), the RL (0-100 cm), root biomass, RLD, and R:S ratio was the highest with HD-3249 under WW (**Figures 3A–D**). Under DW treatment, HD-3249 has the highest RL and RLD, while it was HD-2967 with the highest root biomass and DBW-187 with the highest R:S ratio. Compared to WW treatment, DW had RL reduction of 30%, 34%, 30%, 20%, and 10% for HD-2967, HD-3086, HD-3226, DBW-187, and HD-3226, respectively. Furthermore, DW treatment of HD-2967, HD-3086, HD-3226, DBW-187, and HD-3226 had reduction in root biomass (8%, 46%, 48%, 17%, and 13%, respectively) compared to WW treatment. In addition, the RLD under the DW was 14-33% less than the WW treatment. However, the R:S ratio of HD-2967, DBW-187 and HD-3226 was greater under the DW than WW, while HD-3086 and HD-3249 had greater R:S ratio under the WW than the DW treatment.

### Leaf area and leaf relative water content

**Figure 4:**
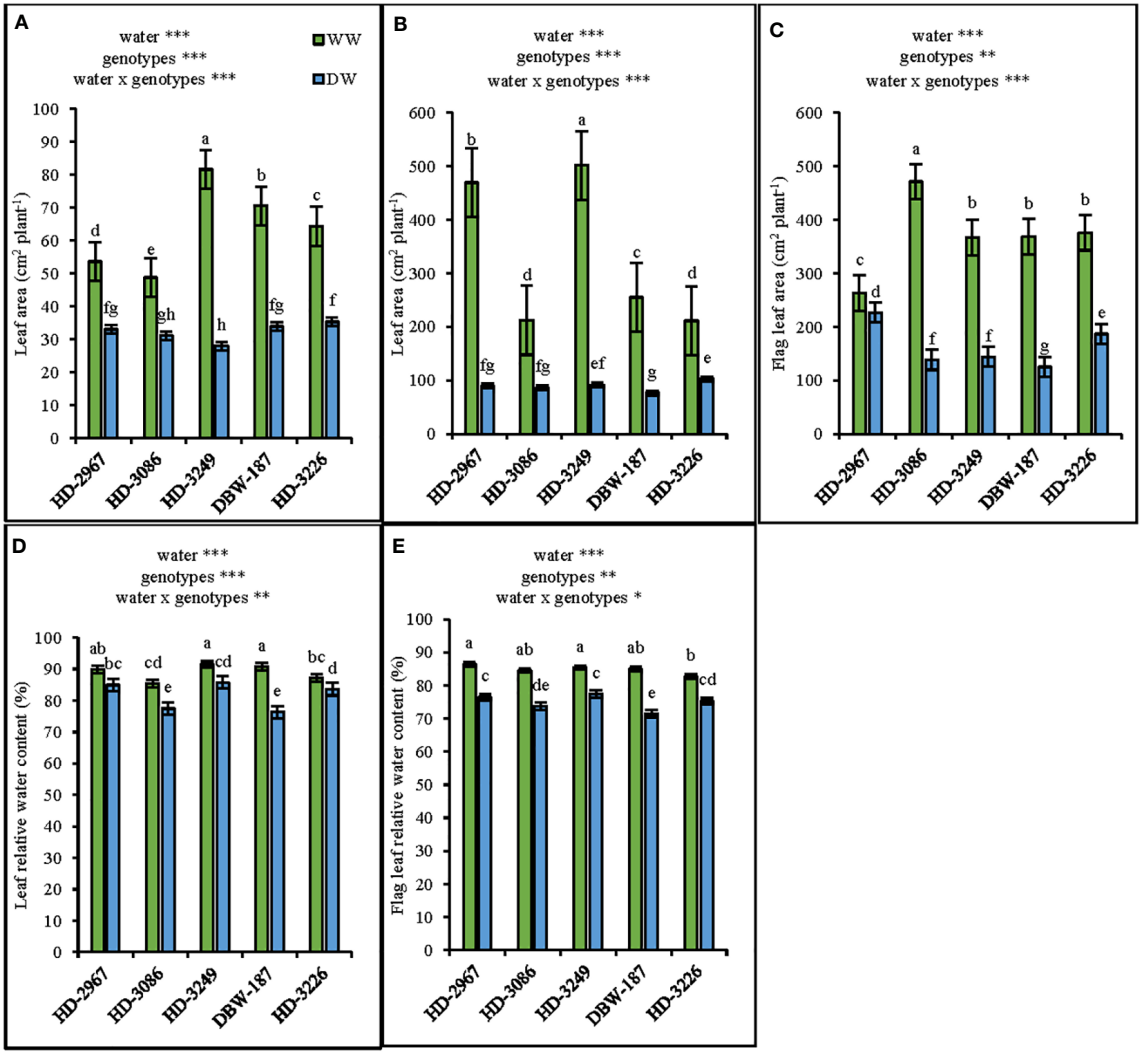
Leaf area and leaf relative water content of five wheat genotypes under well- (WW) and deficit-(DW) watered conditions. **(A)**: leaf area at 45 days (Z25) **(B)**: leaf area at flowering (Z61), **(C)**: flag leaf area at flowering (Z61), **(D)**: leaf relative water content at flowering (Z61), and **(E)**: flag leaf relative water content at flowering (Z61). Letters above the bars indicates significant difference among genotypes at p<0.05 (Turkey’s HSD test). Significant at *0.05. **0.01, and ***0.001 probability level.

The leaf area at 45 d (Z_25_) under the WW treatment was the highest with HD-3249, and significantly greater than the other genotypes (p>0.001). Subsequently, under the DW, HD-3226 resulted in the highest leaf area which was similar to others except HD-3249. The increment in leaf area under WW over DW was 38, 36, 65, 51, and 45% for HD-2967, HD-3086, HD-3249, DBW-187, and HD-3226 (**Figure 4A**). Similarly, the highest leaf area (excluding flag leaf) at flowering (Z_61_) under the WW was produced with HD-3249, while it was HD-3226 under DW (**Figure 4B**). In contrast, HD-3086 and HD-2967 recorded significantly greater flag leaf area under the WW and DW, respectively (**Figure 4C**). Indeed, the flag leaf area was greater than leaf area under the DW, and the contrast holds true for the WW. The leaf relative water content under the WW for HD-2967, HD-3086, HD-3249, DBW-187, and HD-3226 was 5%, 9%, 6%, 15%, and 4% higher compared with their corresponding leaf relative water content under the DW (**Figure 4D**). Furthermore, the flag leaf relative water content under the WW was the highest with HD-2967, while under the DW it was HD-3249 (**Figure 4E**). Across the genotypes, the flag leaf relative water content under the WW was 8-15% greater over the DW (p<0.001).

### Shoot biomass

The shoot biomass of different plant parts at the flowering (Z_61_) and maturity (Z_89_) varied largely among the genotypes and water treatment (**Table 2**). At flowering, HD-3249 under the WW had the highest biomass viz., stem, leaf, flag leaf, and spike, while the least was recorded with HD-3086. In contrast, genotype HD-2967 under the DW resulted in the highest biomass production except in flag leaf, and HD-3086 had the least biomass. There was an increment of 30%, 42%, 44%, 46%, and 39% for total shoot biomass with HD-2967, HD-3086, HD-3249, DBW-187, and HD-3226, respectively under the WW than in DW. Similarly, the biomass at maturity of different plant parts under the WW was the highest with HD-3249 except the flag leaf (the highest with HD-2967). HD-2967 under the DW produced the highest leaf and total shoot biomass, while the highest stem and flag leaf biomass were recorded with HD-3226 and HD-3249, respectively. HD-2967, HD-3086, HD-3249, DBW-187, and HD-3226 under WW gave 30, 43, 45, 53, and 32% more total shoot biomass than the DW treatment.

**Table 2:** Shoot biomass of vegetative organs at flowering (Z_61_) and maturity (Z_89_) of wheat genotypes grown under well- and deficit-watered conditions.

Treatment		DM at flowering (g plant^-1^)					DM at maturity (g plant^-1^)				
		Stem	Leaves	Flag leaf	Spike	Total	Stem	Leaves	Flag leaf	Spike	Total
WW	HD-2967	11.7^bc^	3.4^abcd^	1.8^ab^	9.3^ab^	26.2^b^	9.5^bc^	2.3^abc^	1.2^a^	8.6^a^	21.6^b^
	HD-3086	10.4^cd^	2.4^bcde^	1.2^abc^	7.4^bc^	21.4^c^	8.8^bcd^	1.5^abcd^	0.7^ab^	6.9^ab^	17.9^c^
	HD-3249	14.5^a^	4.5^a^	1.9^a^	10.1^a^	30.9^a^	11.9^a^	2.8^a^	1.0^ab^	9.1^a^	24.7^a^
	DBW-187	12.9^ab^	3.6^abc^	1.7^ab^	8.0^abc^	26.2^b^	10.8^ab^	2.6^ab^	0.9^ab^	7.0^ab^	21.4^b^
	HD-3226	11.4^bc^	3.8^ab^	1.7^ab^	9.5^ab^	26.4^b^	9.0^bcd^	2.5^ab^	1.0^ab^	8.7^a^	21.3^b^
DW	HD-2967	8.5d^e^	2.0^cde^	1.2^abc^	6.4^cd^	18.2^d^	7.2^def^	1.3^bcd^	0.7^ab^	5.9^bc^	15.1^d^
	HD-3086	5.9^f^	1.3^e^	0.7^c^	4.4^d^	12.3^f^	5.0^f^	0.7^d^	0.4^b^	3.9^cd^	10.1^e^
	HD-3249	7.9^ef^	1.9^cde^	1.4^abc^	5.9^cd^	17.2^d^	6.2^ef^	1.0^cd^	0.8^ab^	5.3^bcd^	13.4^d^
	DBW-187	6.5^ef^	1.6^e^	0.9^bc^	5.1^d^	14.0^ef^	5.0^f^	0.9^cd^	0.7^ab^	3.4^d^	9.9^e^
	HD-3226	8.3^de^	1.8^de^	0.9^bc^	5.1^d^	16.1^de^	8.0^cde^	1.0^cd^	0.6^ab^	4.6^cd^	14.3^d^
Water		***	***	***	***	***	***	***	***	***	***
Genotype		**	*	***	**	***	*	*	**	**	***
Water × genotype		*	*	ns	ns	*	**	ns	ns	ns	*

Data are the mean of three replicates for each genotype and water treatments. Values in a column followed by the different letters are significantly different at p<0.05 as determined by Tukey’s honestly significant difference test among the varieties. DM is dry matter and spike at maturity is glumes + awns without grain. For ANOVA results, *p<0.05, **p<0.01, ***p<0.001, and ns not significant.

### Biomass remobilisation and contribution to grain biomass

The remobilised biomass of different plant parts under both the water treatments was the highest with HD-3249, except in spike under the DW with DBW-187 (**Table 3**). The genotype HD-3249 remobilised significantly greater total shoot biomass under the WW than all other genotypes (p<0.001). Similarly, under DW, HD-3249 remobilised the highest total shoot biomass, though it was comparable with HD-2967, DBW-187, and HD-3226 but significantly higher than HD-3086. The total shoot biomass remobilised under the WW was 32, 37, 39, 35, and 34% higher than the DW for HD-2967, HD-3086, HD-3249, DBW-187, and HD-3226 (p<0.001).The contribution of remobilised biomass to grain biomass varied among the genotypes and water treatment. Under the WW treatment, stem biomass contribution was the highest with HD-2967 and being comparable with other genotypes (except HD-3086). HD-3249 had the highest leaf biomass contribution, while HD-3249 and DBW-187 had similar flag leaf biomass contribution. The biomass contribution by the spike was the highest with DBW-187, wherein it was comparable to HD-3249, but being significantly greater than other genotypes. Further, under DW treatment, DBW-187 resulted in the highest stem and spike biomass contribution, while it was HD-3226 for leaf biomass and HD-2967 for flag leaf biomass, respectively. The total contribution under both the water treatments was the highest with HD-3249 (WW) and similar to HD-2967 (DW), however, 6-24% greater than the other genotypes and water treatment.

**Table 3:** Dry matter remobilisation of the vegetative organs and contribution to grain dry weight of wheat genotypes grown under well- and deficit-watered conditions.

Treatment		DMR (g plant^-1^)					Contribution of DMR to grain yield (%)				
		Stem	Leave	Flag leaf	Spikes	Total	Stem	Leave	Flag leaf	Spikes	Total
WW	HD-2967	2.2^ab^	1.1^ab^	0.6^ab^	0.6^ab^	4.6^bc^	17.5^a^	9.0^ab^	4.7^abc^	4.9^bc^	36.1^bc^
	HD-3086	1.7^abc^	0.9^b^	0.5^ab^	0.5^ab^	3.5^cd^	14.5^cd^	8.4^b^	4.2^bcd^	4.0^c^	31.1^fg^
	HD-3249	2.7^a^	1.7^a^	0.9^a^	1.0^a^	6.3^a^	16.7^abc^	10.5^a^	5.4^ab^	6.1^abc^	38.6^a^
	DBW-187	2.1^ab^	1.1^ab^	0.7^ab^	0.9^ab^	4.8^b^	15.7^abc^	7.9^b^	5.4^ab^	7.2^a^	36.2^bc^
	HD-3226	1.7^abc^	1.3^ab^	0.7^ab^	0.7^ab^	4.4^bc^	15.4^abc^	8.7^ab^	4.7^abc^	4.8^bc^	33.5^de^
DW	HD-2967	1.2^bc^	0.7^b^	0.5^ab^	0.5^ab^	3.1^de^	15.9^abc^	8.9^ab^	6.1^a^	6.8^ab^	37.8^ab^
	HD-3086	0.9^c^	0.6^b^	0.2^b^	0.4^b^	2.2^e^	12.3^d^	8.7^ab^	3.1^cde^	5.3^abc^	29.3^g^
	HD-3249	1.7^abc^	0.9^b^	0.6^ab^	0.6^ab^	3.8^bcd^	15.8^abc^	8.6^ab^	5.3^ab^	5.9^abc^	35.5^cd^
	DBW-187	1.5^bc^	0.7^b^	0.2^b^	0.7^ab^	3.1^de^	16.8^ab^	8.1^b^	2.1^e^	7.4^a^	34.5^cde^
	HD-3226	1.3^bc^	0.8^b^	0.3^b^	0.5^ab^	2.9^de^	15.1^bc^	9.1^ab^	2.9^de^	6.0^abc^	33.2^ef^
Water		***	***	***	***	***	ns	ns	***	*	**
Genotype		***	***	**	***	***	***	*	***	**	***
Water × genotype		ns	*	ns	ns	*	ns	ns	***	ns	**

Data are the mean of three replicates for each genotype and water treatments. Values in a column followed by the different letters are significantly different at P<0.05 as determined by Tukey’s honestly significant difference test among the varieties. DMR is dry matter remobilised and spike at maturity is glumes + awns without grain. For ANOVA results, *p<0.05, **p<0.01, ***p<0.001, and ns not significant.

### Nitrogen content

There was a large variation in the nitrogen (N) content between water treatments and genotypes at flowering (Z_61_) and maturity (Z_89_) stages (**Table 4**). The N content under WW at Z_61_ in root, stem, leaf, and spike was the highest with HD-3249, which was significantly greater than the other genotypes, except HD-2967 for spike. HD-2967 had the highest N content in the flag leaf and being comparable to HD-3249 and HD-3226, but significantly greater than HD-3086 and DBW-187. Under the DW treatment, HD-2967 had the highest N content in root, leaf, and spike, while it was HD-3249 for stem and flag leaf. Furthermore, under the WW at Z_89_, HD-2967 had the most N content in stem and flag leaf, while the highest N content in leaf and grain was recorded with HD-3249, and HD-3226 for spike N content. Under the DW, N content in stem, leaf, and spike was the highest with HD-2967, while it was HD-3249 with the highest N content in the flag leaf and grain. There was a reduction of 50, 48, 44, 43, and 49% grain N content under the DW for HD-2967, HD-3086, HD-3249, DBW-187, and HD-3226 compared to the WW treatment.

**Table 4:** Nitrogen content at flowering (Z_61_) and maturity (Z_89_) of wheat genotypes grown under well- and deficit-watered conditions.

Treatment		N content at flowering (mg plant^-1^)					N content at maturity (mg plant^-1^)				
		Root	Stem	Leave	Flag leaf	Spike	Stem	Leave	Flag leaf	Spike	Grain
WW	HD-2967	23.5^de^	65.6^c^	38.8^c^	27.3^a^	41.8^ab^	20.6^a^	10.6^b^	6.4^a^	17.5^b^	163.6^b^
	HD-3086	32.8^b^	53.5^d^	25.7^d^	20.9^b^	32.8^c^	14.1^c^	6.0^c^	2.6^cd^	16.5^b^	131.1^c^
	HD-3249	39.9^a^	83.4^a^	56.0^a^	25.2^a^	44.1^a^	15.8^bc^	14.8^a^	5.0^ab^	19.6^b^	197.2^a^
	DBW-187	30.0^bc^	72.6^b^	38.0^c^	21.7^b^	29.9^c^	17.4^b^	11.3^b^	4.6^abc^	11.3^c^	161.9^b^
	HD-3226	26.9^cd^	63.4^c^	44.8^b^	25.4^a^	40.0^b^	14.5^bc^	13.3^ab^	4.4^abc^	23.8^a^	160.9^b^
DW	HD-2967	21.6^ef^	30.5^ef^	19.6^e^	13.4^d^	19.5^d^	10.6^d^	6.2^c^	2.4^cd^	11.1^c^	81.4^f^
	HD-3086	13.5^h^	20.4^g^	10.8^g^	6.0^f^	16.0^d^	3.2^f^	1.7^de^	0.9^d^	8.1^cde^	67.9^g^
	HD-3249	18.5^fg^	33.7^e^	17.7^ef^	16.5^c^	18.5^d^	3.5^f^	1.2^e^	2.9^bcd^	6.3^e^	109.0^d^
	DBW-187	16.9^gh^	27.8^f^	14.3^fg^	9.8^e^	16.9^d^	3.5^f^	2.2^de^	1.8^d^	7.1^de^	91.5^e^
	HD-3226	14.3^h^	29.1^f^	15.6^ef^	11.1^de^	17.6^d^	7.4^e^	4.3^cd^	1.1^d^	9.9^cd^	81.1^f^
Water		***	***	***	***	***	***	***	***	***	***
Genotype		***	***	***	***	***	***	***	**	***	***
Water × genotype		***	**	***	*	**	ns	***	ns	**	*

Data are the mean of three replicates for each genotype and water treatments. Values in a column followed by the different letters are significantly different at p<0.05 as determined by Tukey’s honestly significant difference test among the varieties. Spike at maturity is glumes + awns without grain. For ANOVA results, *p<0.05, **p<0.01, ***p<0.001, and ns not significant.

### Nitrogen remobilisation and contribution to grain N

The remobilised N under the WW treatment in stem and leaf was significantly greater with HD-3249, which was 33, 41, 18, 20% and 31, 52, 35, 23% more than HD-2967, HD-3086, DBW-187, and HD-3226 (**Table 5**). The highest N remobilised in flag leaf was recorded in HD-3226, being similar to the other genotypes, but significantly greater than DBW-187. HD-3249 and HD-3226 had similar remobilised N in spike, but being significantly greater than HD-3086, HD-3226, and DBW-187. The total N remobilised was the highest in HD-3249, wherein it was 22, 38, 23, and 20% greater than HD-2967, HD-3086, DBW-187, and HD-3226. Under the DW treatment, HD-3249 had the highest remobilised N in different plant parts (stem, leaf, flag leaf, and spike) (**Table 5**). HD-3249 had 27, 45, 25, and 29% more total N remobilised compared to HD-2967, HD-3086, DBW-187, and HD-3226. The remobilised N contribution to the grain N under the WW treatment through stem and leaf was the highest in HD-3249, while it was HD-3226 for the flag leaf and HD-2967 for the spike. The total contribution was 2-8% greater with HD-3249 over the other genotypes. Under the DW treatment, the highest stem remobilised N contribution was recorded with HD-3249, while HD-2967 resulted in the highest remobilised N contribution for the leaf and flag leaf, and HD-3086 for the spike. The total remobilised N contribution was the highest with HD-3249, while the least was recorded with HD-3086 (**Table 5**).

**Table 5:** Nitrogen remobilisation and contribution to grain nitrogen of the vegetative organs of wheat genotypes grown under well- and deficit-watered conditions.

Treatment		N_R_ (mg plant^-1^)					N_R_ contribution to grain N (%)				
		Stem	Leave	Flag leaf	Spike	Total	Stem	Leave	Flag leaf	Spike	Total
WW	HD-2967	45.0^c^	28.2^bc^	20.9^a^	24.2^a^	118.3^bc^	27.7^bc^	17.0^bc^	12.7^abc^	15.0^a^	72.3^b^
	HD-3086	39.4^d^	19.7^d^	18.3^ab^	16.3^b^	93.7^d^	30.3^ab^	15.0^cd^	14.0^a^	12.3^b^	71.3^b^
	HD-3249	67.6^a^	41.2^a^	20.2^ab^	24.4^a^	153.5^a^	34.3^a^	20.7^a^	10.0^cde^	12.3^b^	78.0^a^
	DBW-187	55.2^b^	26.7^c^	17.2^b^	18.6^b^	117.8^c^	34.0^a^	16.7^bcd^	10.7^bcd^	11.7^bcd^	72.7^b^
	HD-3226	53.9^b^	31.6^b^	21.0^a^	16.2^b^	122.6^b^	33.3^a^	19.7^ab^	13.3^ab^	10.0^de^	76.0^ab^
DW	HD-2967	19.8^gh^	13.4^ef^	11.0^cd^	8.4^d^	52.7^f^	24.3^c^	16.3^bcd^	13.7^a^	10.3^cde^	65.3^c^
	HD-3086	17.2^h^	9.1^g^	5.1^e^	7.9^d^	39.3^g^	25.0^c^	13.7^cd^	7.3^e^	12.0^bc^	57.7^e^
	HD-3249	30.2^e^	16.5^de^	13.6^c^	12.2^c^	72.5^e^	27.7^bc^	15.0^cd^	12.7^abc^	11.0^bcde^	66.3^c^
	DBW-187	24.3^f^	12.1^fg^	8.1^de^	9.8^cd^	54.2^f^	27.0^bc^	13.3^d^	9.0^de^	11.0^bcde^	60.0^de^
	HD-3226	21.7^fg^	11.3^fg^	10.0^d^	7.7^d^	50.8^f^	27.0^bc^	14.0^cd^	12.3^abc^	9.7^e^	62.7^cd^
Water		***	***	***	***	***	***	***	*	**	***
Genotype		***	***	***	***	***	***	**	***	**	**
Water × genotype		*	**	*	*	**	ns	*	***	*	ns

Data are the mean of three replicates for each genotype and water treatments. Values in a column followed by the different letters are significantly different at p<0.05 as determined by Tukey’s honestly significant difference test among the varieties. N_R_ is N remobilised and spike at maturity is glumes + awns without grain. For ANOVA results, *p<0.05, **p<0.01, ***p<0.001, and ns not significant.

### Water use efficiency

**Figure 5:**
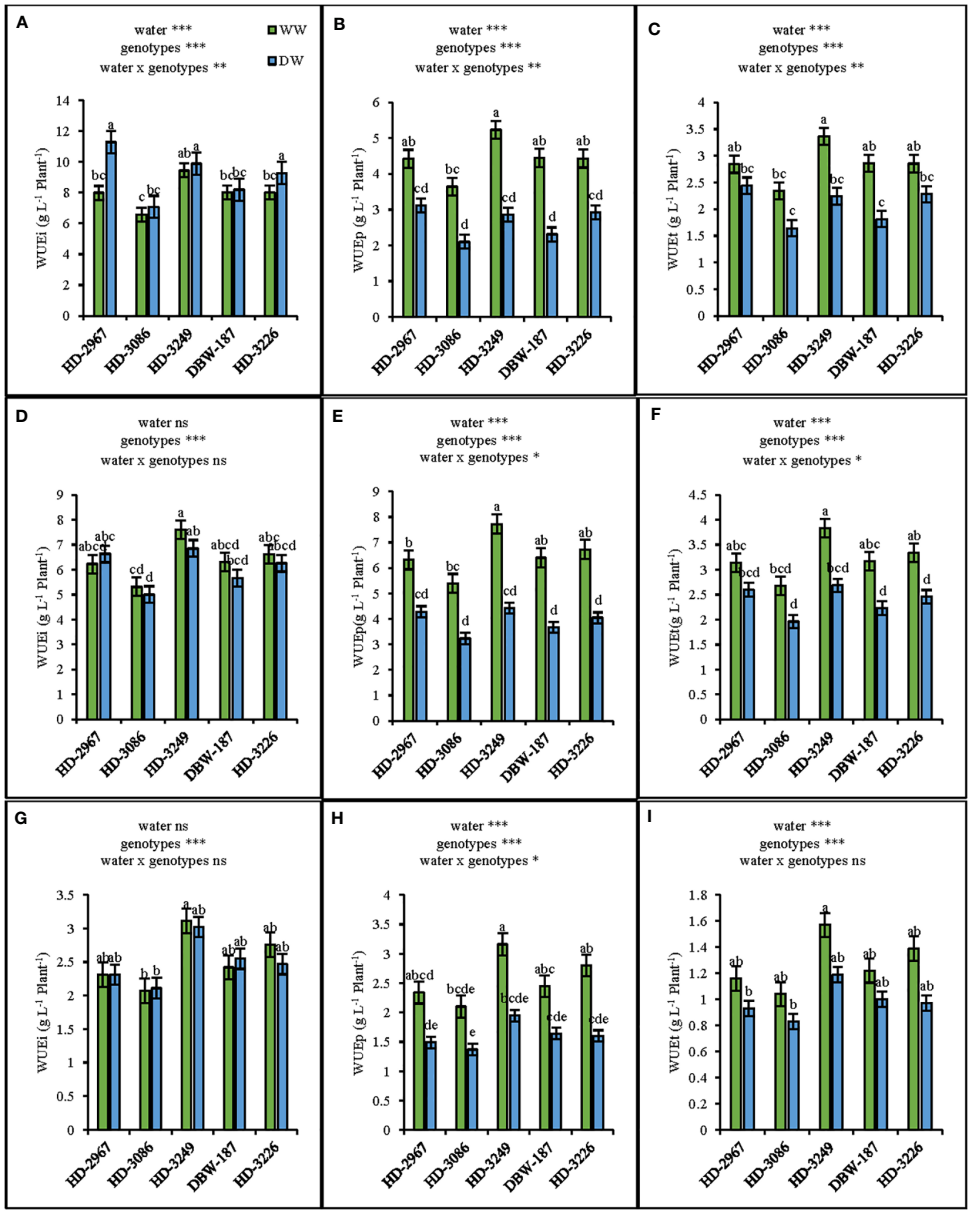
Water use efficiencies of five wheat genotypes at flowering (Z61) and maturity (Z89) grown under wel-(WW) and deficit (DW) watered conditions. Dry matter use-efficiency at flowering **(A)**: irrigation water use efficiency (WUEi) **(B)**: precipitation water use efficiency (WUEp); **(C)**: total water use efficiency (WUEt) Dry matter water use-efficiency at maturity (excluding grain) **(D)**: irrigation water use-efficiency (WUEi) **(E)**: precipitation water use efficiency(WUEp); **(F)**: total water use efficiency (WUEt), Grain water use efficiency **(G)**: irrigation water use efficiency (WUEi) **(H)**: precipitation water use efficiemcy (WUEp) **(I)**: total water use efficiency (WUEt). Letters above the bars indicates significant difference among genotypes at p<0.05 (Turkey;s HSD test). Significant at *0.05, **0.01. and ***0.001 probability levels.

The water use efficiency (WUE) at flowering (Z_61_) under the well-watered (WW) treatment was the highest with HD-3249 and comparable with the other genotypes except HD-3086 (**Figures 5A-C**). While, under the deficit-watered (DW) condition, HD-2967 resulted in the highest WUE, wherein it was similar to HD-3249 and HD-3226, but being significantly greater than HD-3086 and DBW-187 (p<0.001). The irrigation WUE under the DW was significantly greater than the WW (p<0.001), but the total WUE under the WW resulted in 14-36% higher values than the DW across the genotypes. Similarly, at maturity (Z_89_), the highest WUE was recorded with HD-3249, whereby it was significantly greater than HD-3086 under the WW, but the genotypes were comparable under the DW (**Figures 5D-F**). Compared with DW, grain total WUE of HD-2967, HD-3086, HD-3249, DBW-187, and HD-3226 in WW increased by 19%, 20%, 24%, 18%, and 30%, respectively (**Figures 5G–I**).

### Nutrient uptake

**Figure 6:**
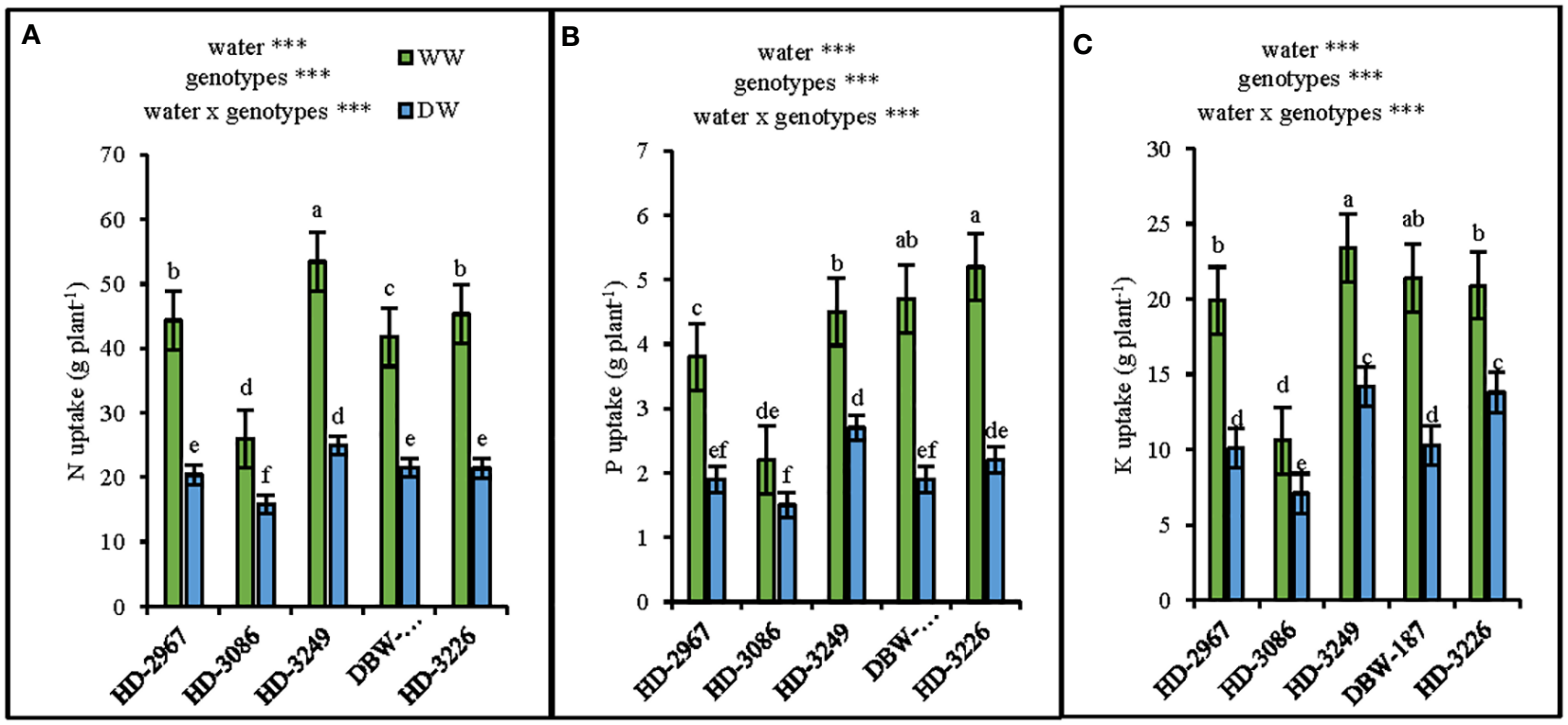
Nutrients uptake of five wheat genotypes at maturity (Z89) grown under well- (WW) and deficit (DW) watered conditions. **(A)**: Nitrogen uptake, **(B)**: Phosphorus uptake, and **(C)**: Potassium uptake. Letters above the bars indicates significant difference among genotypes at p<0.05 (Turkey;s HSD test) Significant at *0.02, **0.01, and ***0.001 probability levels.

At Z_89_, the N uptake under WW treatment for HD-2967, HD-3086, HD-3249, DBW-187, and HD-3226 increased by 53%, 38%, 53%, 48%, and 52%, respectively over the DW treatment (**Figure 6A**). The highest N uptake was obtained with HD-3249 in both the water treatments. The highest phosphorous uptake under the WW treatment was recorded with HD-3226, however, in DW it was HD-3249, while the uptake among the genotypes was enhanced by 31-59% under the WW over the DW treatment (**Figure 6B**). HD-3249 had the highest potassium (K) uptake in both WW and DW, with the least uptake recorded with HD-3086 under the DW treatment. There was an increment of K uptake by 49, 33, 39, 51, and 33% for HD-2967, HD-3086, HD-3249, DBW-187, and HD-3226 under the WW over the DW treatment (**Figure 6C**).

### Correlation coefficients

The shoot biomass at flowering and harvest had significant association with the grain yield (p<0.05) under the WW (**Supplementary Table 1A**), while the biomass remobilised under the DW had higher and significant association with the grain yield compared to the WW (**Supplementary Table 1B**). The RL and RLD under DW had stronger correlation with the grain yield over the WW. The total NR under both the water treatment had significant association with the grain yield (p<0.01). The dry matter accumulation at flowering and harvest under the WW treatment had strong and significant association with the grain WUE (p<0.05) (**Supplementary Table 1A**). The coefficients between grain WUE and RL, grain WUE and RLD were higher under the DW compared to the WW (**Supplementary Table 1B**).

## Discussion

Irrigation regimes had a great impact on the growth and development of plants, whereby, the optimum water supply enhanced the yield potential and productivity. The present study from the PVC-tubes found that the genotypes under the well-watered (WW) condition significantly increased the grain yield over the deficit-watered (DW) condition. The increment of grain yield under the well-watered was 37%, while among the genotypes it ranged between 33-42% (**Table 1**). Grain yield in wheat and biomass at maturity have been reported to be directly related, wherein irrigation treatments with higher biomass at maturity results in greater grain yield compared to no irrigation (Reynolds et al., 2012; Xu et al., 2018). The shoot biomass at maturity in this study under WW was 41% higher compared to DW treatments (**Table 2**). Further, the higher grain yield of HD-3249 could be attributed to the greater number of grains spike^-1^ and the thousand grain weight (TGW), while in HD-3226 to a greater number of the effective tillers plant^-1^, which are the important yield components in wheat responsible for increasing the grain yield (Hall and Richards, 2013; Fang et al., 2017). The reduced grain yield under deficit-watered could be the result of lesser effective tillers, leaf area and photosynthetic activities coupled with the enhanced leaf senescence (Yang et al., 2000). In addition, water stress during the early grain filling in wheat have been reported to shorten the grain filling period and curtail the sink capacity (Saini and Westgate, 1999). The improvement in grain yield and greater stress tolerance has a positive association, resulting from the selection for higher yield stability (Tollenaar and Lee, 2002), as yield stability entails better drought tolerance than the yield potential (Simane et al., 1993). Among the genotypes, the reduction in grain yield of DBW-187 (33% reduction) under DW was comparatively lower, hence could be exploited for dry areas with limited irrigation, while the genotype HD-3249 could well be suited for irrigated condition. The increased grain yield under well-watered conditions might be the result of split nitrogen application (three splits under well-watered against the two under the deficit-watered), leading to a better N uptake and partitioning within the plant system. Subsequently, influencing the growth and development of the crop, as the response of wheat to N fertilization relies on the availability of water (Tilling et al., 2007). Therefore, the findings from this study further confirmed the importance of irrigation is prominent for continued enhancement of grain yield in wheat.

Root systems play a vital role in the crop improvement under the limited water environments (Fang et al., 2017). Wheat genotypes with a vigorous root system during the early growth stage and having greater root mass and root length density had the advantage of capturing more water (Palta et al., 2011), thereby facilitating the better growth with the enhanced leaf area development and shoot biomass (Liao et al., 2006). The genotype HD-3249 with higher root (**Figure 2E**) and shoot biomass (**Figure 2D**) coupled with a greater leaf area (**Figure 4A**) at 45 d (Z_25_) and flowering (Z_61_) under well-watered conditions could be considered as an early vigor genotype. Further, the growth and development of root system is closely associated with wheat grain yield (Wang et al., 2014), wherein greater root biomass significantly enhanced the shoot biomass, which contributes to higher grain yield (Zhang et al, 2009). In the present study, the genotypes under well-watered have significantly greater root (**Figures 2D, E**, **3B**) and shoot biomass, and subsequently the grain yield (**Tables 1**, **2**) compared to the deficit-watered condition. Irrigation was also found to increase the root length density (RLD) of wheat in the 0-80 cm soil layer, compared to non-irrigation treatment (Qin et al., 2004). In this study, the well-watered conditions increased the RLD by 33% and 27% at 45 d (0.0-50 cm) and flowering (0.0-100 cm), respectively over the deficit-watered treatment (**Figure 3C**). Correlations were analyzed between root length, RLD, grain yield and other components in the present study. The results show that grain yield was positively associated with root length and RLD (**Supplementary 1A, B**), and stronger association was observed under the DW compared to the WW, thereby implies the importance of genotype with better root system in limited water conditions. The interaction between water and N has been found to confer complementary effects on the growth of wheat root and above-ground plant parts (Li et al., 2004). In the current study, though the N application rate was similar in both the treatments, WW result in significantly higher root growth (root length, RLD, root biomass) at Z_25_ (**Figures 2A, B, E**) and Z_61_ (**Figures 3A–C**) stage of the crop coupled with greater shoot biomass (**Figure 2D**; **Table 2**). Furthermore, WW treatment with better root system had 4-15% and 8-15% more leaf and flag leaf relative water content respectively, which could be attributed to higher soil water status (Ozkur et al., 2009) in addition to greater extraction of water from the deeper soil layers. It further substantiates in this study, whereby genotype HD-3249 with greater root length and root biomass have relatively higher leaf and flag leaf RWC under both the water treatments (**Figures 4D, E**).

The carbon needed for grain filling in wheat depends on the current photosynthesis and remobilization of reserves stored in the stem and other plant parts (Pheloung and Siddique, 1991). The dry matter accumulated at flowering and maturity under the WW had significant positive correlation with the grain yield (**Supplementary 1A**), while the amount of dry matter remobilized was positively correlated with the grain yield in both the water treatments (**Supplementary 1A, B**). The capacity of stem reserves under the drought stress gets reduced due to lesser photosynthesis, thereby reducing the availability of photosynthates for further storage (Zhang et al., 2017) coupled with the reduction in the mobilized stem reserves (Ehdaie et al., 2006). In this study, the stem dry matter at anthesis under the deficit-watered decreased by 39% compared to the well-watered treatment, while the whole plant dry matter reduced by 40% (**Table 2**). Further, the amount of dry matter remobilized and its contribution to grain yield under the deficit-watered decreased by 36% and 3%, respectively; which could be attributed to the lesser dry matter production at anthesis (Ehdaie et al., 2008; Zhang et al., 2017). In addition, the well-watered treatments led to a better root growth, thereby enhancing the extraction capacity of soil available water and nutrients, resulting in a greater biomass production at the anthesis. Among the genotypes, HD-3249 had the highest contribution of dry matter remobilization to the grain yield under the well-watered, while HD-2967 under the deficit-watered treatments (**Table 3**). The higher dry matter remobilization under the well-watered environment might have helped the plants in maintaining the grain growth by thwarting the reduced photosynthesis post-anthesis (Xue et al., 2006) and eventually improved the grain yield.

The nitrogen (N) accumulated in different plant parts at the anthesis (Z_61_) and maturity (Z_89_) under the well-watered environment was significantly greater than the deficit-watered (**Table 4**). This could be due to the better development of root-system with the enhanced capability of acquiring the available soil N leading to greater uptake and better partitioning within the plant system. Prior studies in the wheat had reported that N- accumulation before anthesis provides a major source of grain N (Gaju et al., 2014), wherein the remobilization of the stored N in roots and shoots before anthesis contributed 50-90% of the grain N (Palta and Fillery, 1995; Kichey et al., 2007). In this study, the contribution of remobilized N from shoots (stem, leaf, flag leaf, and spike) to grain N among the genotypes ranged between 71-78% (WW) and 57-66% (DW), respectively (**Table 5**). The greater contribution under the well-watered condition might be the result of higher N uptake at anthesis (Zhang et al., 2017), due to more splits application of N (three for WW and two for DW).

The most important sources for grain N are the stems and leaves, while the contribution from roots and chaff is about 10% and 15%, respectively (Dalling, 1985). Similar results of a greater contribution from the stem (24-34%) and leaf + flag leaf (22-33%) than that of chaff (9-15%) were recorded in the present study (**Table 5**). The greater N accumulated at the anthesis prolonged the ability of leaf area to be sustained, thereby enhancing the carbon assimilation through higher interception of solar radiation (Fischer et al, 1993), resulting in the improved grain filling rate and grain yield (Zhang et al., 2017). Furthermore, the nutrient uptake at harvest i.e., N, P, and K under the well-watered condition increased by 38-53% (**Figure 6A**), 31-50% (**Figure 6B**), and 33-51% (**Figure 6C**), respectively among the genotypes. The greater uptake could be associated with the improved root system and enhanced the above-ground biomass production, as the soil water status influences the uptake, translocation, and assimilation of nutrients (particularly N in wheat) (Li et al., 2009; Shi et al., 2012).

Water use efficiency (WUE) can be enhanced through the optimal supply of nutrients and a mild soil stress with 65-70% of the field capacity (Wang et al., 2012; Liu et al., 2018). In contrast, Karam et al. (2009) reported that the supplemental irrigation and N rate doesn’t affect the WUE in wheat. In this study, biomass WUEi (irrigation WUE) under the deficit-watered environment at flowering (Z_61_) was 16% greater than to the well-watered (**Figure 5A**), while at maturity (Z_89_) it was comparable between the two water treatments (**Figure 5D**). On an average, the biomass WUEt (irrigation + precipitation) under the well-watered at Z_61_ and Z_89_ was 27% (**Figure 5C**) and 25% (**Figure 5F**) more, respectively over the deficit-watered conditions. The higher WUE under WW could be attributed to more dry matter partitioning to the above-ground biomass with lower root:shoot ratio, as the assimilation product consumed by the root is twice that of the shoots (Zhang et al, 2009; Ma et al., 2010). The root: shoot ratio at flowering in current study was 15% greater under the DW compared to the WW treatments (**Figure 3D**). The grain WUEt was enhanced by 25% (**Figure 5I**) with the well-watered compared to the deficit-watered. Similar finding of the reduced biomass WUE in wheat under the irrigated conditions was reported by Liu et al. (2018). Further, there observed strong positive correlation between WUE and RL/RLD particularly under the DW treatment (**Supplementary 1B**). Nevertheless, the higher WUEt under well-watered could be attributed to a greater biomass production before the anthesis, thereby reduced soil evaporation through better surface area coverage and could offset the loss through transpiration (Wang et al., 2018).

## Conclusion

Deficit-watered (no irrigation at active tillering and only ½ the amount of well-watered at the grain filling stage) reduced the total dry matter and N- accumulation, root-system traits, and finally the grain yield. Among the genotypes, the reduction in grain yield under the deficit-watered was to the tune of 33-42%. Genotype HD-3249 resulted in the highest grain yield under both the well- and deficit-watered condition, but the least reduction was recorded with the genotype DBW-187. The total root length (0.0-100 cm), root biomass and root length density at flowering under the well-watered environment increased by 28%, 30%, and 28%, respectively over the deficit-watered, in contrast, the root: shoot ratio was decreased by 15%. The irrigation WUE at flowering was more under the deficit-watered, but the biomass and grain total WUE was improved with the well-watered condition. These outcomes from the PVC tubes studies suggest that the increase in grain yield and WUE are the results of proper irrigation scheduling and N application coupled with the adoption of genotype suited for a particular environment. Further, detail study on root-shoot relationships under the field conditions could result in better understanding of the genotypic performances in the semi-arid region of the Indo-Gangetic plains.

## Data availability statement

The original contributions presented in the study are included in the article/**Supplementary Material**. Further inquiries can be directed to the corresponding author.

## Author contributions

RZ, NB, and VP, led the research work, planned, supervised, and conducted field experiments, read and edited the manuscript. NB, RZ, KL, AnB, and SB, collected soil/plant samples and performed chemical analysis, also wrote the initial draft of the manuscript, prepared figures, and tables. DK, YS, AD, NK, RP, ArB, RC, and KS, project supervision, reviewed, read and edited the manuscript with significant contributions. RJ and KD performed statistical analysis. All authors contributed to the article and approved the submitted version.
