# Supplementary material for: Above and below-ground growth, accumulated dry matter and nitrogen remobilization of wheat (Triticum aestivum) genotypes grown in PVC tubes under well- and deficit-watered conditions
Source: Front Plant Sci. 2023 Mar 7;14:1087343. doi: 10.3389/fpls.2023.1087343 (PMC10027722; doi:10.3389/fpls.2023.1087343)
Supplement: Supplementary file 1 [file DataSheet_1.docx]

**Supplementary Table 1** Correlation coefficients among grain yield, shoot biomass, root traits at flowering (Z_61_), nitrogen remobilised and water use efficiency; (a) well- and (b) deficit-watered.

| (a) | GY | DMF | DMH | DMR | RL | RLD | RDW | NR | WUE_F_ | WUE_H_ |
| --- | --- | --- | --- | --- | --- | --- | --- | --- | --- | --- |
| DMF | 0.91^*^ |  |  |  |  |  |  |  |  |  |
| DMH | 0.88^*^ | 0.99^**^ |  |  |  |  |  |  |  |  |
| DMR | 0.86 | 0.97^**^ | 0.97^**^ |  |  |  |  |  |  |  |
| RL | 0.56 | 0.77 | 0.80 | 0.84 |  |  |  |  |  |  |
| RLD | 0.59 | 0.80 | 0.83 | 0.87 | 0.99^**^ |  |  |  |  |  |
| RDW | 0.65 | 0.76 | 0.78 | 0.89^*^ | 0.90^*^ | 0.92^*^ |  |  |  |  |
| NR | 0.94^*^ | 0.99^**^ | 0.98^**^ | 0.97^**^ | 0.78 | 0.81 | 0.80 |  |  |  |
| WUE_F_ | 0.90^*^ | 0.99^**^ | 0.99^**^ | 0.98^**^ | 0.78 | 0.81 | 0.78 | 0.99^**^ |  |  |
| WUE_H_ | 0.97^**^ | 0.99^**^ | 0.97^**^ | 0.95^*^ | 0.71 | 0.74 | 0.74 | 0.99^**^ | 0.98^**^ |  |
| WUE_G_ | 1.00^**^ | 0.91^*^ | 0.88^*^ | 0.86 | 0.56 | 0.59 | 0.65 | 0.94^*^ | 0.91^*^ | 0.97^**^ |

| (b) | GY | DMF | DMH | DMR | RL | RLD | RDW | NR | WUE_F_ | WUE_H_ |
| --- | --- | --- | --- | --- | --- | --- | --- | --- | --- | --- |
| DMF | 0.48 |  |  |  |  |  |  |  |  |  |
| DMH | 0.24 | 0.92^*^ |  |  |  |  |  |  |  |  |
| DMR | 0.92^*^ | 0.72 | 0.44 |  |  |  |  |  |  |  |
| RL | 0.87 | 0.83 | 0.60 | 0.98^**^ |  |  |  |  |  |  |
| RLD | 0.81 | 0.84 | 0.61 | 0.94^*^ | 0.98^**^ |  |  |  |  |  |
| RDW | 0.27 | 0.71 | 0.49 | 0.58 | 0.60 | 0.54 |  |  |  |  |
| NR | 0.97^**^ | 0.64 | 0.37 | 0.98^**^ | 0.95^*^ | 0.93^*^ | 0.41 |  |  |  |
| WUE_F_ | 0.37 | 0.98^**^ | 0.98^**^ | 0.60 | 0.73 | 0.72 | 0.65 | 0.51 |  |  |
| WUE_H_ | 0.69 | 0.97^**^ | 0.84 | 0.86 | 0.94^*^ | 0.92 | 0.64 | 0.80 | 0.92^*^ |  |
| WUE_G_ | 0.99^**^ | 0.52 | 0.27 | 0.95^*^ | 0.90^*^ | 0.85 | 0.31 | 0.98^**^ | 0.41 | 0.73 |

GY- grain yield, DMF- shoot biomass at flowering, DMH- shoot biomass at harvest (excluding grain), DMR- dry matter remobilised, RL- total root length, RLD- root-length density, RDW- root dry matter, NR- total nitrogen remobilised, WUEF- water use efficiency at flowering, WUEH- water use efficiency at harvest (excluding grain), WUEG- grain water use efficiency. Significant at * p<0.05, ** p<0.01.

Supplementary fig.1 Monthly mean temperature and total rainfall during the crop growing period of the study area, ICAR-IARI, New Delhi.
